# Transient protein-protein interface prediction: datasets, features, algorithms, and the RAD-T predictor

**DOI:** 10.1186/1471-2105-15-82

**Published:** 2014-03-24

**Authors:** Calem J Bendell, Shalon Liu, Tristan Aumentado-Armstrong, Bogdan Istrate, Paul T Cernek, Samuel Khan, Sergiu Picioreanu, Michael Zhao, Robert A Murgita

**Affiliations:** 1 School of Computer Science, McGill, Montreal, CA; 2 Department of Microbiology and Immunology, McGill, Montreal, CA; 3 Department of Anatomy and Cell Biology, McGill, Montreal, CA; 4 Department of Biology, McGill, Montreal, CA; 5 Department of Mathematics and Statistics, McGill, Montreal, CA; 6 Department of Biology and Computer Science, McGill, Montreal, CA; 7 Department of Physiology, McGill, Montreal, CA

**Keywords:** Machine learning, Protein-protein interaction, Protein-protein interface, Feature selection, Protein datasets, Protein interface identification, Protein prediction scoring

## Abstract

**Background:**

Transient protein-protein interactions (PPIs), which underly most biological processes, are a prime target for therapeutic development. Immense progress has been made towards computational prediction of PPIs using methods such as protein docking and sequence analysis. However, docking generally requires high resolution structures of both of the binding partners and sequence analysis requires that a significant number of recurrent patterns exist for the identification of a potential binding site. Researchers have turned to machine learning to overcome some of the other methods’ restrictions by generalising interface sites with sets of descriptive features. Best practices for dataset generation, features, and learning algorithms have not yet been identified or agreed upon, and an analysis of the overall efficacy of machine learning based PPI predictors is due, in order to highlight potential areas for improvement.

**Results:**

The presence of unknown interaction sites as a result of limited knowledge about protein interactions in the testing set dramatically reduces prediction accuracy. Greater accuracy in labelling the data by enforcing higher interface site rates per domain resulted in an average 44% improvement across multiple machine learning algorithms. A set of 10 biologically unrelated proteins that were consistently predicted on with high accuracy emerged through our analysis. We identify seven features with the most predictive power over multiple datasets and machine learning algorithms. Through our analysis, we created a new predictor, RAD-T, that outperforms existing non-structurally specializing machine learning protein interface predictors, with an average 59% increase in MCC score on a dataset with a high number of interactions.

**Conclusion:**

Current methods of evaluating machine-learning based PPI predictors tend to undervalue their performance, which may be artificially decreased by the presence of un-identified interaction sites. Changes to predictors’ training sets will be integral to the future progress of interface prediction by machine learning methods. We reveal the need for a larger test set of well studied proteins or domain-specific scoring algorithms to compensate for poor interaction site identification on proteins in general.

## Background

Protein-Protein Interactions (PPIs) occur when two or more proteins bind together, triggering the proteins’ biological activities. With alterations in PPIs being a contributing factor in much of human disease, both academia and industry pursue further understanding of these sites. PPIs can be divided broadly into two types: obligate interactions, where constituents are not stable structures in physiological conditions unless they are in a complex; and transient interactions, where binding partners may dissociate from each other and exist as stable entities in the unbound state [1]. Prediction of obligate interfaces is pharmacologically less interesting since the formation of such interfaces does not typically contribute to signalling cascades. In contrast, the binding of transiently interacting partners almost always leads to important cellular signalling events [1].

PPIs can be viewed at multiple levels: the structural level, in which the atomic details of a molecular interaction are accurately known, and at the cellular network level, which provides a broader overview of the functional relationships and downstream effects of interactions between the proteins in the cell. Protein interaction networks are useful sources of biological information for numerous purposes, including the provision of putative interactions between domains as inferred from known PPIs, such as those derived from two-hybrid screening [2]. While protein networks can provide a more holistic understanding of the processes within the cell, pharmaceutical development relies on a comprehensive understanding of the intricacies of a given interaction, so as to chemically manipulate its function in a precise manner. As such, PPI prediction places emphasis on the more fine-grained, structural approach to elucidating the mechanism of pharmacologically relevant interactions.

Statistical models for understanding and predicting interaction sites largely began with Jones and Thornton in 1997 [3] with their systematic characterisation of PPIs; following, a number of groups have added and refined their initial characterizations, observing PPI sites to be highly conserved, hydrophobic, planar, and protruding [4-9]. As no single feature is sufficient for accurate identification of a PPI site, methods such as linear regression [10] or scoring algorithms modelled after empirical energy functions [11] were employed in early predictors. Although their conceptual simplicity is suited for initial exploration by virtue of a clearer set of results to analyse, these methods require prior knowledge about the features used to characterize the interaction sites, usually in the form of hard-coded parameters. Consequently, these parameters are more prone to bias and incapable of adaptation to new data without direct human intervention. Researchers are increasingly turning to machine learning (ML), frequently using algorithms such as support vector machines (SVM) [12,13], neural networks [14-16], Bayesian networks [17-19], and random forests [20].

Accurate ML prediction relies on sets of features, used to distinguish instances of a given class, being consistent across query and training instances. For training data, the typical PPI predictor uses either a very small, hand-selected set of well-studied proteins [12,21,22] or a large number of proteins obtained through automated filtering of an online protein structure database [13,23] such as the Protein Data Bank (PDB) [24]. This is generally accompanied by a small set of characterising features and a supervised learning algorithm [12,18,21,25-29].

We present an analysis of machine learning protein interface prediction (MLPIP), inspecting the process from beginning to end. We investigated a large set of interface residue features drawn from previous efforts, a large PPI dataset filtered from the PDB, and numerous machine learning algorithms, including logistic regression, decision trees, and Bayesian networks. From this analysis we achieved a predictor that scored higher than competing predictors using a well labelled training dataset of proteins with a decision tree learning algorithm and four characterising features.

## Methods

### Training and testing dataset

In an attempt to exclude proteins participating in obligate interactions, we generated our training set by selecting only proteins which can form stable structures *in vivo* without needing to be bound to other proteins [30]; crystal structures of obligate interactions should not satisfy this criteria. We only took proteins with structural deposits in the PDB as it is a necessary requirement for the analysis of structural feature that contribute to PPIs. Many intrinsically disordered proteins (IDPs), which lack stable tertiary structures, also participate in transient PPIs. The lack of PDB entries of IDPs limits their use in PPI prediction. The training set consisted of a collection of proteins having only one entry in the COMPND record of the PDB, representing proteins in an unbound state so they could be queried and trained in their native conformations. Protein files with a single COMPND entry most often contain multiple, nearly identical, chain entries representing either subunits of a homomultimer or similar conformations of the same protein included for completeness; these files represent the monomers in the training set.

To control the quality and variety of the monomers in the training set, proteins were filtered by properties in their PDB files. These included structure resolution (≤3.5 Å), number of chains (≤5), chain length (≥100 and ≤800), and chain length standard deviation (the difference between the multiple chains of the same compound listed in the PDB file was ≤5), ensuring proteins in the set were of high quality and regular size, as well as ensuring regular handling of chain heads and tails. The number of connections (≤1000) and number of disulphide bonds (≤100) were filtered to remove unusual proteins such as 2IWV, a monomeric porin laced with ligands, and 3V83, a transport protein with a high number of disulphide bridges. In addition, antigen-antibody complexes were removed and the presence of nucleotides in the protein structure was disallowed to restrict training samples to strictly protein-protein interactions. We also mandated that crystallographic B-factors be given for all atoms so that the values could be used directly as a feature by machine learning.

With the above criteria applied to a 2013 January snapshot of the PDB, the monomer dataset is reduced to 48,424 structures. The majority of protein exclusion was due to chain length restrictions, as many smaller entities in the PDB are not proteins at all but are instead fragments or pharmacological compounds. Lastly, proteins with more than 70% sequence identity, as determined by BLAST [31], were considered duplicates, and only one was kept. This was done to avoid the over-representation of proteins whose structure has been recorded many times, though some homologues may remain in the dataset. Of each remaining structure, the chain used to represent the monomer was the longest chain in the PDB file or the chain with the lowest sum of B-factors (should there be several chains of the same length). If no chains had a lower sum of B-factors or longest length, then the first chain in the file was used.

A protein complex was defined as a structure having more than one COMPND entry. To identify which monomers in the dataset have interaction partners, each monomer was aligned to all chains of all protein complexes in the PDB. A match was found if there was at least 80% sequence identity between one of the chains in the complex and the monomer. A geometric definition of a PPI was adopted for the identification of interacting residues using a distance cut-off between chains. For a residue to be considered part of an interacting site, it was required that there be at least one pair of atoms at a distance of at most 4.5 Å between two interacting chains.

Interface mapping successfully mapped 663 monomers to complexes. After PDB validation and feature calculations, 392 monomers remained, with many discarded proteins representing complexes without acceptable interfaces as judged by our program. An unacceptable interface site was most commonly found on a complex where there were no atoms within 4.5 Å between two “interacting” chains or a site that had less than 90% residue identity between the complex and monomer representations of the chain (usually due to a poorly resolved residue), which did not allow us to exactly map the interacting residues directly back to the monomer.

The monomer-complex interface mapping process described is effective for complexes stored directly in the pdb file, but ignores the biologically relevant multimeric form as predicted by the crystallographers (stored in the BIOMT field of the pdb files). For all proteins in the training set, the BIOMT data was analysed and interface mapping was performed, treating monomer files, as well as the complexes to which they were mapped, with the appropriate BIOMT information, thereby creating new interfaces for use in machine learning. Biologically, these are important to preserve, but they made no statistically significant difference to the performance of the predictor on the dataset as measured by cross validation. Most importantly, we aimed to maintain consistency with datasets used by other learners derived from data mining, and as such, these interface sites are not included in the below data, but data and results for most tests below is available in the supplemental material (Additional file 1: Table S5).

#### Improving data labelling

It must be addressed that a protein with a low percentage of observed interacting residues is likely not a result of lack of interactions, but rather a lack of complexes by which to identify the interacting residues of the protein. Thus, the use of proteins with unknown or unidentified interactions, which can be a problem even in manually curated datasets, leads to a systematic increase in type II error. As it is impossible to guarantee knowing every interface site for a given protein, an approach of assuming better identification of these sites in proteins with higher rates of identified interacting residues was adopted. We considered a protein as divisions of 100 residue domains, as suggested by the typical size of a domain [32], and assumed the accuracy of residue labelling was quite good if there was at least one interface site per domain (based on observations of protein domain sizes and interaction site distribution [33,34]), and comparatively better if there were at least two interface sites per domain. This reduced the possibility of missing interfaces, though potentially not fully eliminating it. To thoroughly explore the effect of data labelling accuracy, two testing subsets were generated from the full training set: a set with proteins where there is at least one interface per 100 residues (NI1) and a set of proteins where there are at least two interfaces per 100 residues (NI2).

#### Compensating for class imbalance

In total, only a small fraction of all residues in the full training set were identified as interacting, creating a class imbalance problem in MLPIP where the number of non-interacting residues far outweighs the number of interacting ones, resulting in a high bias toward the majority class [35-37]. The issue was explored with random under-sampling, where instances of the majority class are removed until a target distribution of each class is attained. To find the optimal distribution, a set of proteins was repeatedly under-sampled to produce training sets with the proportion of interacting residues ranging from 30% to 70% of the training instances. These were then compiled, trained upon, and cross-validated for their true positive rates and true negative rates to find the point at which these rates met for a balanced prediction.

### Machine learning features

Features used to predict interface sites were categorised as either residue features or residue-local features. To calculate residue-local features, a residue was considered with each of its neighbours, a set typically ranging from two to six residues on which calculations were performed over their collective surface area with the resulting value applied to the nodal residue. The use of residue-local features allows the calculation of the basic geometric attributes: protrusion, roughness, surface density, and curvature. The scope of these features is limited so that the integrity of the residue value is little affected by the values of residues further away on the protein. A baseline set of residue features was chosen from the pool of commonly-used features in MLPIP: these were relative solvent-excluded surface area (relSESA), B-factors, hydrophobicity, propensity, electrostatic potential, energy of solvation, conservation, evolutionary rate-shift, and disorder. For details on the calculations of each of these features, please refer to Additional file 2. To ensure proper citation of tools used to calculate some of these features, we note that MSMS [38] was used to calculate Solvent Excluded Surface Area as defined by Connolly [39], the mean maximum residue surface area was calculated using UCSF Chimera [40] (Additional file 3: Table S1); the CX algorithm [41] was used to calculate protrusion; roughness was calculated as a function of protein surfaces, as recommended by Pettit and Bowie [42]; the scale by Fauchére and Pliska [43] was used to calculate hydrophobicity; electrostatic potential was derived with APBS [44]; the curvature calculation was modelled on an approach by Coleman et al. [45]; rate shift was computed with the Rate4Site algorithm [46]; and conservation was given by the ScoreCons algorithm [47]. These tools are commonly used in MLPIP studies and were here used to give values as similar as possible to previous efforts for individual attributes.

### Machine learning algorithms

The Weka software package [48] was used for all the machine learning algorithms, where default parameters were employed for the following algorithms: Logistic Regression, Multilayer Perceptron, Bayesian Networks, and a variety of decision tree models. In particular, an Alternating Decision-Tree model, which specialises in two-class problems and performed very well in testing, was used for the final predictor. The classifiers were validated using leave-one-out cross-validation (LOOCV). Instead of leaving out a single residue, which is regularly the instance, each cross-validation leaves out a full protein, including all of its constituent residue instances. Classifiers were trained on a training set preprocessed to remove all instances of one particular test protein and tested on the residues belonging to the protein removed. This is iterated over the entire set of training proteins.

### Statistical analysis of results

Prediction of interacting site was evaluated and measured using the following statistics:

(1) $$\begin{array}{ll} \text{TPr} & =\frac{\text{TP}}{\text{TP}+\text{FN}}\; \end{array}$$

(2) $$\begin{array}{ll} \text{TNr} & =\frac{\text{TN}}{\text{TN}+\text{FP}}\; \end{array}$$

(3) $$\begin{array}{ll} \text{PRC} & =\frac{\text{TP}}{\text{TP}+\text{FP}}\; \end{array}$$

(4) $$\begin{array}{ll} \text{MCC} & =\frac{\left(\text{TP}\times \text{TN})-(\text{FP}\times \text{FN}\right)}{\sqrt{\left(\text{TP}+\text{FN})(\text{TP}+\text{FP})(\text{TN}+\text{FP})(\text{TN}+\text{FN}\right)}}\; \end{array}$$

(5) $$\begin{array}{ll} F1 & =2\frac{\text{PRC}\times \text{TPr}}{\text{PRC}+\text{TPr}}\; \end{array}$$

Where TP is the number of true positives, TN is true negatives, FP is false positives, and FN is false negatives. The statistics calculated here are: true positive rate (TPr), also commonly referred to as sensitivity or recall, true negative rate (TNr), also known as specificity, precision (PRC), the Matthews correlation coefficient (MCC), and F measure (F1). Following LOOCV, mean MCC is given as an average score over a set of proteins. Pearson’s correlation between features and interaction site involvement was computed using MATLAB and evaluated using a two-tailed test of significance. Statistics of machine learning results were tested with the Wilcoxon signed-rank test using R, where the difference in score was ranked by absolute value and each rank was modified by the sign of the difference, i.e. whether the change was positive or negative after treatment. The modified ranks were then summed to generate the test statistic *W*, for which the sampling distribution approaches normality and a p-value can be calculated based on the z-score.

### Feature set selection

Feature set selection was performed across machine learning algorithms and training sets, using cross-validation to select optimal models based on MCC scores from LOOCV. A genetic algorithm was used to produce and intelligently evolve attribute sets, which were then evaluated by a variant of the RACE algorithm [49], in which all individuals of a given generation were raced against an elite (i.e. a best feature set yet found) of the previous generations. Computational efficiency was further supplemented by a variant of the Hoeffding Race algorithm described by Maron and Moore [50] with a simple addition, replacing the highest confidence bounded average $x_{\text{max}}=\bar{x}+\varepsilon$ with a weighted average of the current empirical mean and *x*_*max*_, representing the unevaluated data, to take advantage of the known data set size.

## Results and discussion

### Datasets

The full feature set was calculated for every residue regardless of whether it was exposed on the surface or buried within the core of the protein, since buried residues may become exposed during interactions and thus play a role or affect nearby surface residues and modify their ability to participate in interactions. Within the total testing set of 392 proteins, an average of 13% of residues were labelled as interacting. However, there was wide deviation (see Additional file 4: Table S2), with, for example, 1WYY having nearly all of its residues labelled interacting. An unusually high proportion of interacting residues, here considered greater than 50%, occurred in only 9 proteins. The NI1 test set of 71 proteins with at least 1 interface per 100 residues, which likely has fewer unidentified interaction sites, has 26% of its residues labelled interacting, and NI2, a test set of 15 proteins used for comparative data with existing servers with at least 2 interfaces per 100 residues, has 36% of its residues labelled interacting.

Since it is of the general consensus that manually identified interaction sites from published sources generate a more accurate dataset, a thorough analysis of more than 80 publications on more than 70 protein complexes and their protomers was performed. We found that papers reporting on the complexes often do not identify the interaction site completely, instead identifying only a few residues, which the authors considered to be of interest. Papers that provided a more complete description of interaction sites proved almost identical to interaction sites mined by our program when 72 manually selected proteins and their interface sites were examined (data not shown). Our automated method provides similarly accurate information regarding interaction sites, but with vastly improved efficiency over manual sifting of publications.

Other avenues of exploration are available to PPI prediction, such as the investigation of integrating protein network information into transient PPI prediction models. This potentially includes novel feature extraction and the creation of PPI datasets via putative interface databases. Conversely, PPI prediction may provide structural detail on the characteristics of the interactions present in protein networks. A comparison of datasets derived from the putative interaction networks versus those calculated from structural information could provide insight into the relevant characteristics of PPIs, as well as a mutual and independent validation of the features discovered via both methods.

### Optimal class distribution

**Figure 1:**
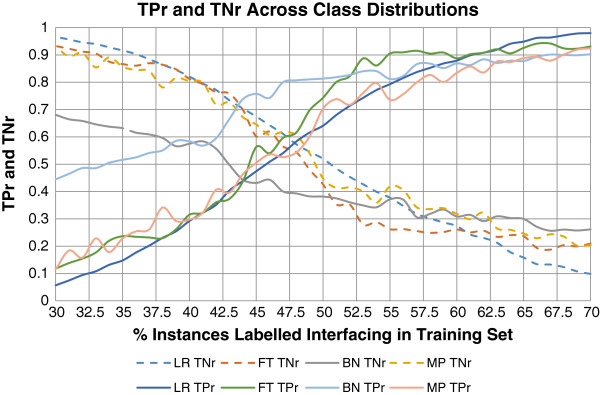
The true negative (TNr) and true positive rates (TPr) for Logistic Regression (LR), Baysian Network (BN), Multilayer Perceptron (MP), Functional Tree (FT), various classifiers depending on the proportion of instances labelled as interacting as evaluated by LOOCV on the total test set, where the true negative rates are universally descending and the true positive rates universally ascending.

By varying the proportion of interacting and non-interacting residues in the training set, we observed that the ability of the classifier to predict a class is strongly influenced by the proportion of instances of that class present in the training set (Figure 1). The point at which the classifier was able to predict positively and negatively equally well was consistent across most classifiers, with 45%-50% of residues labelled positively interacting. The exception was the Naive Bayes classifier, which predicted optimally with approximately 40% interacting residues and 60% non-interacting.

### Feature set evaluation

An analysis of each feature’s correlation to positive labelling was made using the Pearson product-moment correlation coefficient (Additional file 5: Table S3). The geometric features – surface area, protrusion, and roughness – exhibited the greatest significant correlation with interaction site presence; the features hydrophobicity, residue propensity, solvation energy, B-factors, and electrostatic potential displayed reduced yet significant correlation. The remaining features, rate shift, curvature, conservation score, and disorder demonstrated poor correlation with weaker statistical significance (*P* > 0.001).

Though features with the highest correlation to protein interface presence are most desirable, machine learning classifiers rely on the collective effect of the features involved, where those best correlating with interaction site are not necessarily the best features with which to train due to orthogonality and other concerns. For testing the reliability of each feature in predicting interaction sites and to take advantage of the effect of combinations of features, we used a modified genetic search algorithm to find optimal feature sets for a variety of evaluation classifiers (Table 1).

**Table 1:** The features selected by the iterative genetic search algorithm with evaluation by Logistic Regression (LR), Baysian Network (BN), Functional Tree (FT), REP Tree (RT), and Alternating Decision Tree (AT)

**Feature**	**Count**	**LR**	**BN**	**FT**	**RT**	**AT**
relSESA	10	□∙	□∙	□∙	□∙	□∙
esolv	6	□	□∙	∙		□∙
Density	6	∙		∙	□∙	□∙
ePot	5		□∙	□	□	∙
Scorecons	5	□∙	∙	□	□	
rate4site	5	□	∙	∙	□	
Disorder	5	□∙		∙	□	□
B-Factor	4		∙	□∙		□
Roughness	4	□∙	□∙			
Hydro	3	∙		□	□	
Protrusion	3	□∙			□	
Propensity	3	□		□	∙	
Curvature	2		□∙			

A white box (□) represents the feature was selected when the algorithm was tested on all proteins, using leave-one-out cross-validation, while a feature with a black circle (∙) was selected when tested on the NI1 subset. Count is the number of times a feature was selected by either datasets tested.

The best correlated features by absolute value of raw correlation with positive labelling were relSESA (0.188), protrusion (0.180), roughness (0.153), and density (0.088). However, some of these features also have high correlation with each other, reducing their utility; protrusion and relSESA have a pairwise correlation of 0.88 and relSESA and roughness have a correlation of.329 with each other. Feature poorly correlated with positive labelling include conservation, rate shift, and disorder, each having less than 0.02 correlation.

Multilayer Perceptrons were excluded from our feature selection analysis due to excessive run-time required to generate the optimal feature set, which yielded insignificant increases in score. While it is expected that different classifiers will place varying value on distinct attributes, we found surface area, solvation energy and density to be consistently selected. Further, electrostatic potential, conservation, rate shift (which is another measure of conservation), and disorder were also frequently present amongst the resulting optimal feature sets, chosen by at least 3 out of 5 classifiers.

Assessing the effect of the optimal feature set (Table 2) on both the full protein set and the NI1 subset led to significant improvement in performance for all predictors tested, as determined by the Wilcoxon’s signed rank test (*P* < 0.05 in all cases). In most cases, applying feature selection led to significant improvement in sensitivity (TPr) without improving specificity (TNr). For two classifiers, Baysian Networks and AD-Trees, the change in MCC was not significant after applying feature selection when used on the full protein set, but high significance levels (*P* < 0.001) were reached when applied on the more accurately labelled NI1 subset.

**Table 2:** **F1, MCC, TPr, TNr, PRC using the full set of features (*α*) or only the features selected (*β*) in Table **1

	**LR**						**AT**					
	**Full set**			**NI1**			**Full set**			**NI1**		
	*α*	*β*		*α*	*β*		*α*	*β*		*α*	*β*	
MCC	0.1247	0.1304	*	0.1684	0.188	***	0.1376	0.1418		0.1859	0.2012	***
TPr	0.6462	0.6497		0.6449	0.6597	*	0.7064	0.7615	***	0.7958	0.8047	
TNr	0.5386	0.5435	**	0.5435	0.5519		0.4915	0.4428		0.4049	0.4149	***
PRC	0.2082	0.2108	*	0.3445	0.3543	***	0.2092	0.2053		0.3328	0.3383	***
F1	0.2946	0.2971		0.4359	0.447	**	0.3019	0.304		0.4562	0.4635	***
	**BN**						**FT**					
	**Full set**			**NI1**			**Full set**			**NI1**		
	*α*	*β*		*α*	*β*		*α*	*β*		*α*	*β*	
MCC	0.1356	0.1412		0.188	0.2043	**	0.1319	0.1408	**	0.1607	0.1981	***
TPr	0.7794	0.8553	***	0.7866	0.842	***	0.704	0.7545	***	0.6931	0.821	***
TNr	0.4143	0.3341		0.4197	0.3749		0.4881	0.4504		0.485	0.3905	
PRC	0.2008	.1976		0.3358	0.3341		0.2059	0.2055		0.335	0.3343	
F1	0.3005	0.3007		0.4574	0.4654	**	0.2983	0.3034	***	0.4385	0.4628	***
	**RT**											
	**Full set**			**NI1**								
	*α*	*β*		*α*	*β*							
MCC	0.1059	0.1196	***	0.128	0.1769	***						
TPr	0.6333	0.6583	***	0.6246	0.6986	***						
TNr	0.5227	0.5173		0.5221	0.502							
PRC	0.1999	0.2041	**	0.327	0.343	***						
F1	0.2843	0.2922	***	0.417	0.4461	***						

Algorithm abbreviations in Table 1. The algorithms were applied to the full protein set (Full Set) and the NI1 subset. Statistical significance was calculated using using 1-sided Wilcoxon’s signed rank test. (* *P* < 0.05; ** *P* < 0.01; *** *P* < 0.001).

### Training set evaluation

The ability to accurately identify residues with respect to their involvement in PPIs, which has not been noted in most MLPIP papers, is an issue that is very challenging to solve entirely as it is difficult to ensure that a protein has every interaction site identified. A comparison of prediction accuracy between the full dataset and the NI1 subset showed that regardless of whether feature selection was applied, prediction accuracy as determined by MCC and F measure was significantly improved for all machine learning algorithms (Table 3). The NI1 showed an average of 0.06 increase in MCC using the optimal feature set, representing a 44% increase in performance.

**Table 3:** **Change in MCC and F1 of predictions on the full set of 392 proteins and the better labelled NI1 subset of 71 proteins for various machine learning algorithms (shown in Table **2**) with or without feature selection**

	**Without feature selection**						**With feature selection**					
	***▵*** **MCC**	**%** ***▵*** **MCC**		***▵*** **F1**	**%** ***▵*** **F1**		***▵*** **MCC**	**%** ***▵*** **MCC**		***▵*** **F1**	**%** ***▵*** **F1**	
LR	0.044	35.106	***	0.141	47.960	***	0.058	44. 183	***	0.150	50.43	***
BN	0.052	38.682	***	0.157	52.233	***	0.063	44.726	***	0.165	54.78	***
FT	0.029	21.798	*	0.140	47.035	***	0.057	40.724	***	0.160	52.48	***
RT	0.022	20.943	*	0.133	46.655	***	0.057	47.932	***	0.154	52.67	***
AT	0.048	35.068	***	0.154	51.107	***	0.059	41.926	***	0.158	52.05	***
avg	0.039	30.319		0.145	48.998		0.059	43.898		0.157	52.48	

Algorithm abbreviations in Table 1. Statistical significance was calculated using 1-sided Wilcoxon’s signed rank test. (* *P* < 0.05; ** ***P***< 0.01; *** P < 0.001).

**Figure 2:**
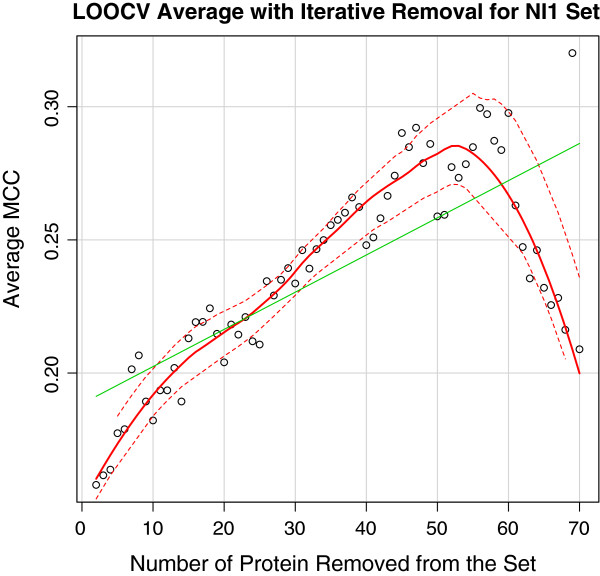
The average MCC calculated by leave-one-out cross-validation as the lowest scoring proteins were iteratively removed from the full dataset.

**Figure 3:**
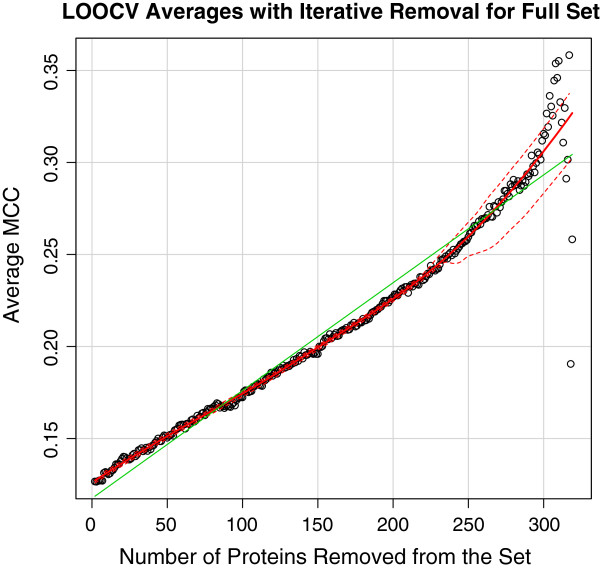
The average MCC for the 10 best scoring proteins calculated by leave-one-out cross-validation as the lowest scoring proteins were iteratively removed from the full dataset.

**Figure 4:**
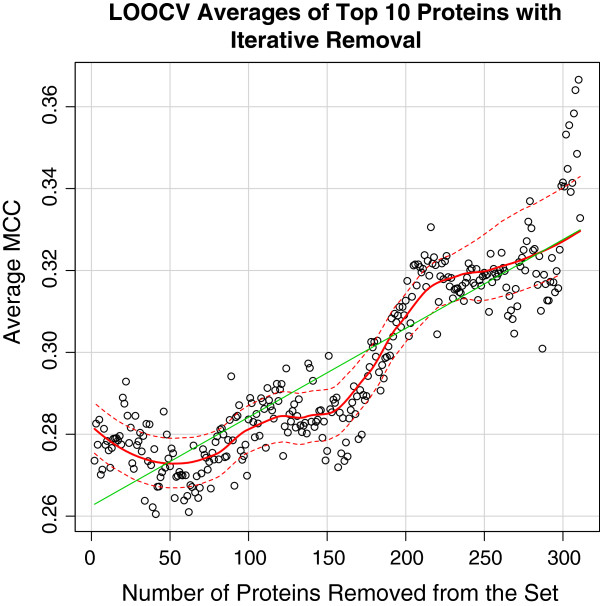
The average MCC calculated by leave-one-out cross-validation as the lowest scoring proteins were iteratively removed from the NI1 dataset.

To reduce noise in the dataset, we performed an iterative removal of proteins that scored the lowest from the training set in cross validation using AD-Trees for the NI1 set and logistic regression for the full training set (Figures 2, and 3), assuming that they were the ones adding the most noise. Expectedly, this resulted in a considerable increase in score. To assess if this is a result of the trend in the moving average of MCCs by the iterative removal of the lowest scoring proteins, we tracked the changes in MCC of the set of 10 proteins (1C9Q, 1I9E, 1J1J, 1MZ4, 1YGT, 1ZKZ, 2JAY, 2R2Y, 3R3Q, 3RKI) that had the highest MCCs when trained with the full protein set. Through the use of a better labelled set to perform the same operation, considerably less variation in score occurs (Figure 4).

When dissected, the effect on score as the worst scoring proteins are iteratively removed is hypothesized to be the combined result of three factors: presence of unknown interaction sites, training set optimisation, and the "odd protein" effect. The odd protein effect refers to proteins that deviate significantly from a typical protein structure, which would here be considered a mix of secondary structures in a moderately globular structure. An example of an “odd protein” is 3MTT, which was the second lowest scoring protein, consisting of only two alpha helices. Another example is 1WYY, a membrane fusion protein with 84% of its residues interacting that is made up of tightly, circularly packed alpha helices, and could not be filtered through basic restrictions. This was the only protein that was removed by hand after discovering its unusual number of interacting residues and highly unusual shape. Testing on the worst 10 proteins that were removed, Promate [18], cons-PPISP [51], PINUP [52], and PIER [28] achieved average MCC scores of.007,.050,.042, and.11 respectively (data not shown), substantially lower than their scores on the full test set (Section ‘A new MLPIP: RAD-T (Residues on Alternating Decision-Trees)’), suggesting this is not a weakness of the predictor used, but a property of the proteins themselves.

After the removal of many proteins with low labelling accuracy and unusual characteristics, training set optimisation begins to occur, where the training set converges on a set of proteins that predict well on each other, but fails to generalise to the majority of the other proteins in the full set. In the case of the full training set, this began when 160 proteins remained. As the training set becomes increasingly small, the performance of the classifier becomes highly variable, which is expected when there are not enough samples for the machine to generalise, resulting in overfitting to the training data and poor performance on the test data.

Increasing the bias in the learning decreased the ability for the machine to generalise on novel data. When testing on the full set using non-feature-selected AD-Trees, training on the top 10 scoring proteins gave an average MCC of 0.085, training on the top 20 gave an average of 0.105, training on the NI1 gave an average of 0.131. LOOCV on the full set gives an average MCC of 0.138. This inability to generalise is not a disadvantage, assuming training sets can be intelligently biased, as many therapeutic applications require accurate prediction on only a few target proteins. For example, identifying proteins with similar structure and using these to specialize the machine or another learning mechanism has been used successfully by PredUS [53] and by PrISE [54].

Unexpectedly, the top scoring proteins demonstrated by our curve had little structural identity with one another, according to DeepAlign [55], PDBeFold [56] and MISTRAL [57]; they also lacked significant functional similarities as judged by their Gene Ontology (GO) tags (see Additional file 2, section two). These proteins’ feature identity with one another as related to their interacting sites has not yet been traced to structural or functional origins. There was no significantly increased Pearson’s correlation between each feature and positive labelling (see Additional file 6: Table S4 for full results). As such, there is certainly some other underlying connection given that these proteins score well against each other within such a small set, with unusual similarity between the properties per residue defining interacting and non-interacting residues. The identification of some biological similarity may enable its application to those proteins that are lower scoring on the curve and could be pursued. We continue to explore this problem in an attempt to better understand what makes a residue’s interaction predictable.

### A new MLPIP: RAD-T (Residues on Alternating Decision-Trees)

To assess the capabilities of existing machine learning protein interface site predictors and to compare their performance to an optimised predictor with a better filtered training set, we created a new predictor, Residues on Alternating Decision Trees (RAD-T). The AD-Tree algorithm was used as the machine learner, having consistently provided the highest scores in Section ‘Feature set evaluation’ and offering potential for optimisation. RAD-T was run with 10 boosting iterations, trained on the NI1 set of proteins, and tested on a modified Docking Benchmark 3.0 set of 188 proteins (DS188) [53,58] to generate a comparable test of performance against other machine learning predictors. Reports of scores from competing programs Cons-PPISP, Meta-PPISP [59], PINUP and Promate were generated previously [54] and used directly to compare to RAD-T. The features used were relSESA, solvation energy, electrostatic potential, and density, chosen by a round of attribute selection with AD-Trees using the NI1 set. Compared to our competitors, at 65% (Table 4), RAD-T had the highest true positive rate, which measures the recall/sensitivity of interacting residues in the prediction. This boost in recall came at the cost of the precision and accuracy of the overall prediction. From an application point of view in the development of therapeutic targets, it is preferable to make an overestimate of the number of residues interacting than to miss a potentially important cluster of interacting residues.

**Table 4:** **Performance measures for RAD-T on Docking Benchmark of 129 proteins, resulting in 188 unbound complexes compared to other servers, including Cons-PPISP (Cons-P), PINUP, Promate, and Meta-PPISP (Meta-P), data for which were previously generated [**[54]**]**

	**RAD-T**	**Cons-P**	**Meta-P**	**PINUP**	**Promate**
TPr	0.647	0.306	0.267	0.347	0.303
PRC	0.285	0.465	0.49	0.407	0.365
F1	0.355	0.369	0.346	0.375	0.331
MCC	0.222	0.267	0.262	0.246	0.195

Since the DS188 set consisted of 14% interacting sites, which is similar to our the full set of proteins, a rate we consider suboptimal, we also tested RAD-T on the NI2 set. The greatest concern for performance testing in this case was overfitting, which can be avoided if all testing proteins are excluded from the training set. However, since the 15 test proteins constitute approximate one-fifth of the training set of 71 proteins, removing all of them strongly skews the learner and would not create an accurate representation of predictor performance. A solution was reached by removing only the query protein from the training set for each test, as in cross validation. While the testing set’s labelling is presumed to be quite accurate and it is unlikely there are many unknown interfacing sites that could artificially decrease scores, this testing set consists only of a small set of proteins and thus needs to be expanded for wider use. RAD-T outperforms its counterparts with a median increase in MCC of 0.11 and average of 59.11%, and an average increase in F measure of 28% (Table 5). The difference in level of performance as compared to the DS188 is in line with our expectation that machine learning performance improves with more accurately labelled datasets. It is notable that 3 out of the 5 competing servers showed a decrease in MCC when tested on the NI2 set compared to the DS188. This is mainly due to the low recall, depicted as TPr, on the part of their servers while TPr for RAD-T improved from 65% to over 80% with the better test data. The size of the testing set is also too small to conduct significance tests that have meaningful interpretations. This difference in score may actually be higher than measured here, as the competitors’ predictors have the distinct advantage of having included our test proteins in their training sets.

**Table 5:** Comparative data for RAD-T and the servers, Cons-PPISP (Cons-P), PINUP, Promate, PIER, and Meta-PPISP (Meta-P)

	**RAD-T**	**Cons-P**	**Meta-P**	**PINUP**	**Promate**	**PIER**	**Average**	**Median**
MCC	0.264	0.147	0.166	0.151	0.136	0.230	0.166	0.151
TPr	0.809	0.322	0.255	0.285	0.939	0.836	0.527	0.322
TNr	0.458	0.810	0.879	0.836	0.152	0.377	0.611	0.810
Prc	0.447	0.493	0.547	0.529	0.400	0.441	0.482	0.493
F1	0.576	0.390	0.348	0.370	0.561	0.577	0.449	0.390
△MCC RAD-T	0.000	0.117	0.098	0.113	0.129	0.034	0.098	0.113
% MCC RAD-T	0.00	79.19	58.73	75.26	94.78	14.88	59.11	75.26
△ F1 RAD-T	0.000	0.186	0.228	0.205	0.015	-0.002	0.126	0.186
% △ F1 RAD-T	0.00	47.73	65.51	55.53	2.66	-0.33	28.16	47.73

These servers were chosen for consistency with literature and because they were the prediction servers that demonstrated reliability or were not altogether offline. Averages and medians indicated do not include RAD-T.

## Conclusion

Our results show that the most useful features for machine learning protein interaction site prediction are, in order, relSESA, solvation energy, density, electrostatic potential, conservation, rate shift, and disorder, as found by feature selection across a variety of machine learning algorithms on our datasets. Relative solvent excluded surface area and solvation energy were also critical features when buried residues were excluded from the dataset (data not shown), indicating that the role of relSESA lies beyond its ability to distinguish buried and exposed residues. We found that optimisation of machine learning algorithms and feature selection produced significantly better results than their unoptimised counterparts. However, these differences are overshadowed by the change resulting from varying restrictions on labelling accuracy. Improvements to dataset labelling led to better predictions by most algorithms tested regardless of whether an optimal feature set is used. Therefore, labelling accuracy in machine learning testing sets needs to be better addressed. As such, previous machine learning predictors may perform much better than their original results suggest.

Through iterative removal we identified those proteins that scored worst, middling, and best. We believe that this method can be an important tool to better understand what makes a protein’s interaction sites highly predictable. In cases of neither obviously poor labelling accuracy nor odd structure, it remains unclear what makes a protein interface highly predictable or unpredictable. Surprisingly, the top 10 scoring proteins in our training set scored almost as well when trained with the noise of the poorly labelled proteins as they did when trained with a better labelled training set.

There are existing ways to specialise training sets, most popularly by using training proteins with high structural similarity to the given testing protein. In our study, the best performing training set that does not use the query protein as a training instance was this set of 10 proteins with low structural identity.

We believe in order for MLPIP to make further progress, future focus will need to be directed towards training set generation and specialisation, and the search for larger testing sets with fully identified interface sites for more reliable scoring and significance tests. We also believe that MLPIP, frequently compared to the more specialised and more easily scored docking, may be undervalued due to poor scores by misinterpreting hidden interface sites.

Using our conclusions and experiments to optimise the training set, feature set, and machine learning algorithm, we created a prediction program that scores substantially higher than previous non-structurally specialising machine learning predictors, with an average increase in score of 59% as compared to five other leading predictors. We expect continued improvement upon the acquisition of more proteins in the PDB both for more training instances and a better labelled testing set, as well as upon the application of specialised training sets for each query protein.

Many areas of extraordinary complexity remain difficult to address by MLPIP, such as the timing, knowledge of physiological conditions, and biochemical modifications of proteins, with regards to their effect on interactions. We wish to explore methods of modelling these phenomena as well as exploring protein interaction networks and putative interactions for use in extending the versatility of future PPI predictors.

## Competing interests

The authors declare that they have no competing interests.

## Authors’ contributions

RAM conceived and supervised this project. CJB and SL contributed significantly to software development and organised the tool chain to perform many of the experiments. PTC edited software and created mislabelling data experiments and results. TAA and BI designed and wrote the genetic-RACE algorithm, and ran experiments and analysed data with SP. SP performed preliminary investigation into feature selection. BI and TAA also optimised machine learning algorithms and applications, and generated most of the data for the manuscript. SK carried out class distribution and iterative protein removal experiments, which were then finalised and analysed by MZ. MZ collected and analysed data from similar applications. CJB and SL wrote the initial draft of the manuscript. All authors edited this article. All authors read and approved the final manuscript.

## Supplementary Material

Additional file 1 **Table S5.** BIOMT Data. Result of inclusion of complexes created from BIOMT field of pdb file. Click here for file

Additional file 2 **Supplementary information for transient protein-protein interface prediction: datasets, features, algorithms, and the RAD-T predictor.** This PDF file details the derivation of machine learning features and the mentioned gene ontology experiments. Click here for file

Additional file 3 **Table S1.** NMRSA. The NMRSA values per residue in a GLY-X-GLY tri-peptide. The NMRSA was calculated using UCSF Chimera, averaging surface area values over 5 different secondary structure and 3 different rotamer libraries. Click here for file

Additional file 4 **Table S2.** Data Descriptive Statistics Summary. A summary of the descriptive statistics for the monomer dataset. In the chart, NI is number of interfaces, CL is chain length, NI : CL is the ratio of number of interfaces to chain length, and PA is percent active residues. Click here for file

Additional file 5 **Table S3.** Feature Correlation Matrix. A confusion matrix of correlations between all features. Click here for file

Additional file 6 **Table S4.** Feature Correlation Matrix for Top 10 Scoring Proteins. A confusion matrix of correlations between all features for the top 10 scoring proteins. Click here for file
