# Supplementary material for: Transient protein-protein interface prediction: datasets, features, algorithms, and the RAD-T predictor
Source: BMC Bioinformatics. 2014 Mar 24;15:82. doi: 10.1186/1471-2105-15-82 (PMC4021185; doi:10.1186/1471-2105-15-82)
Supplement: Additional file 2 — Supplementary information for transient protein-protein interface prediction: datasets, features, algorithms, and the RAD-T predictor. This PDF file details the derivation of machine learning features and the mentioned gene ontology experiments. [file 1471-2105-15-82-S2.pdf]

SUPPLEMENTAL

Supplementary Information for Transient Protein-Protein Interface Prediction: Datasets, Features, Algorithms, and the RAD-T Predictor

Calem J Bendell[1†], Shalon Liu[2,5†], Tristan Aumentado-Armstrong[3], Bogdan Istrate[3], Paul T Cernek[4], Samuel Khan[4], Sergiu Picioreanu[6], Michael Zhao[7] and Robert A Murgita[2*]

[*]Correspondence: robert.murgita@mcgill.ca
[2] Department of Microbiology and Immunology, McGill, Montreal, CA
Full list of author information is available at the end of the article
[†]These authors contributed equally

Supplementary Information for Machine Learning Features and Gene Ontology

1 Machine Learning Feature Calculations

1.1 Solvent Excluded Surface Area

Surface area as derived by MSMS [1] was obtained per residue and then divided by the solvent excluded surface area of the mean maximum residue surface area (MMRSA) for the residue's type. The MMRSA was calculated using UCSF Chimera [2], simulating each residue in a GLY-X-GLY tri-peptide and averaging surface area values over 5 different secondary structures and 3 different rotamer libraries (MMRSA values are listed in Supplementary Table S1). If a residue is buried in the protein core, such that it has no exposed surface, it is not included in the machine learning training set.

1.2 Local Protrusion

Local protrusion is defined by a protrusion index multiplied with the accessible surface area of each residue. The protrusion index is calculated for each residue by averaging the atomic protrusion index using the CX algorithm [3], defined as the ratio of unoccupied to occupied volume within a 10 Å radius of the atom. The local protrusion was calculated using the formula:

$$Protrusion = \frac{\sum_i p_i SA_i}{\sum_i SA_i} \quad (1)$$

Where SA_i is the surface area for residue i with a probe radius of p_i.

1.3 Local Roughness

Roughness was calculated as a function of protein surfaces, as recommended by Pettit and Bowie [4], using a variant of their smoothed atomic fractal dimension (SAFD). Three local surface areas, defined by three surface areas over the same residue-local space with different probe radii are computed and the average of all possible combinations of surface pairs is given as the final result. The total

roughness is calculated with:

$$Roughness = \frac{1}{3}\sum_{i\leq j}^{j\leq 3} 2 - \frac{log|SA_i - SA_j|}{log|p_i - p_j|} \tag{2}$$

Where SA_i is the surface area for the residue with a probe radius of p_i.

1.4 Local Surface Density

The local surface density was defined as the number of atoms in a group of residues that are within 2.4 Å of the surface divided by the surface area of involved residues. Division by surface area results in density as a function of surface area, rather than volume, found to be a more relevant calculation.

1.5 B-Factors

B-factors, also known as Debye-Waller Factors or temperature factors, give the probability that an atom spends a particular amount of time in one space, which can be calculated during collection of X-Ray Crystallography data. Though computationally predictable given a protein structure, the values provided in the structures deposited in the PDB were used directly without modification.

1.6 Hydrophobicity

Hydrophobicity is measured as the free energy of transfer between two immiscible liquids. A number of hydrophobicity scales have been developed with a variety of rankings for the twenty standard amino acids. Here the scale provided by Fauchére and Pliska [5] was used.

1.7 Residue Propensity

A number of amino acids have been noted to be preferentially found at interacting sites [6], a feature considered here by residue propensity. Propensity was defined as the relative contribution of a given residue to the interface normalised to the proportions of the residue type in the whole protein, given by the following equation where X is residue type.

$$Propensity = \frac{\frac{\#\ X\ in\ interface}{\#\ X\ in\ whole\ protein}}{\frac{interface\ size}{protein\ size}} \tag{3}$$

1.8 Electrostatic Potential

An electrostatic map was calculated by solving the Poisson-Boltzmann partial differential equation for a protein in an aqueous solution using APBS [7]. The resulting mesh of potential energy was mapped to the surface of each protein from which the surface atom values of each residue were averaged to derive a total electrostatic potential along the surface of the protein per residue.

1.9 Energy of Solvation

Here the solvation energy was calculated using the accessible surface area model according to the equation.

$$Solvation\ Energy = \sum \alpha_i SA_i \tag{4}$$

where SA_i is the surface area of atom i; and α_i is the atomic solvation parameter based on calculations by Fernández-Recio et. al [8].

1.10 Local Curvature

The method for curvature calculation was modeled on an approach by Coleman et al. [9] where curvature is given as the radius of the least-squares fitted sphere to the protein surface, found using geometric inversions of the least-squares fitted plane.

1.11 Residue Conservation and Rate Shift

BLAST was used against all known protein sequences to identify related proteins and a multiple sequence alignment was performed, from which two types of conservation scores were calculated: a rate shift score, defined as the change in rate of evolution of each residue using the Rate4Site algorithm [10] and a conservation score using the ScoreCons algorithm [11].

1.12 Disorder

The RONN [12] algorithm was used for the disorder feature for each residue. Disorder was calculated for individual residues with RONN's default parameters.

RONN, the Regional Order Neural Network, recognises patterns that are common amongst natively disordered regions in proteins and scores the likelihood of disorder against these patterns.

2 Gene Ontology Tag Frequency and Significance

We use the Gene Ontology database to find functional tags of proteins [13]. When looking for functional tags that characterize a given set, we always compare that set to the full set of 392 proteins. The main function of this procedure is to examine sets of proteins and look for patterns in their tags. This is done by comparing the frequencies of tags occurring between two sets of proteins. For instance, set 1 could be a large set of proteins and set 2 could be the subset of set 1 that had the best prediction scores; then, if any tags are significantly more common in set 2 than set 1, such tags could presumably be considered to characterize the high scoring set (i.e. such tags could be used to build functionally targeted training sets for other proteins with the same tags).

This calculation is performed as follows:

1. Obtain all of the gene ontology tags for every protein in the two sets. Note that we only look at "Molecular Function" tags.
2. Designate one set as the "subset" of interest. We will look for characterizing tags of this set. (We call it a subset because this set is generated as a subset of the second set in the majority of cases.)

3 For every tag in the subset, calculate the following statistics:
   (a) Number of appearances of the tag in the subset
   (b) Number of appearances of the tag in the other set
   (c) Frequency of the tag in the subset
   (d) Frequency of the tag in the other set
   (e) Relative frequency of the tag in the subset compared to the other set
   (f) The statistical significance of the frequency difference, using the p-value of the Wilcoxon rank-sum test
4 Use these statistics to look for characterizing patterns in the tags in one set versus the other

We calculate frequency and relative frequency as follows:

$$Frequency\ of\ tag\ in\ set\ A = \frac{Number\ of\ occurences\ in\ set\ A}{Number\ of\ total\ proteins\ in\ set\ A}$$

$$Relative\ Frequency\ of\ tag\ in\ subset\ B = \frac{Frequency\ of\ tag\ in\ subset\ B}{Frequency\ of\ tag\ in\ other\ set\ C}$$

2.1 Criteria for Selecting Characteristic Molecular Function Tags

To choose which tags are characteristic of the subset, a number of criteria must be satisfied. First, the protein must be present in a large fraction of the subset. This implies that the frequency of the tag in the subset must be fairly high; in our case, we required at least 30% of the proteins to possess the tag. Second, the protein must be more common in the subset than in the other (generally parent) set; that is, its relative frequency in the subset must be greater than 1. Third, the difference in relative frequency must be statistically significant (i.e. not likely to happen by chance). As such, we require the likelihood (i.e. the Wilcoxon rank-sum p-value) of the relative frequency occurring under the null hypothesis to be less than 0.05. For tag T in subset A and other set B, we summarize these criteria as follows:

- $Frequency\ of\ T\ in\ A > 0.3$
- $Relative\ Frequency\ of\ T\ in\ A\ versus\ B > 1$
- $P_{Wilcoxon}(A_{dist} = B_{dist}) < 0.05$

3 BIOMT Data

In order to create complexes based on the Biomolecule (BIOMT) field of pdb files, we used the MakeMultimer program (Michael Palmer, University of Waterloo) to perform the transformations suggested by the BIOMT fields of each protein. This allowed us to map the interfaces arising from interactions between multiple instances of a protein.

4 Supplementary Results and Tables

Table S1 The NMRSA values per residue in a GLY-X-GLY tri-peptide. The NMRSA was calculated using UCSF Chimera, averaging surface area values over 5 different secondary structure and 3 different rotamer libraries.

Table S2 A summary of the descriptive statistics for the monomer dataset. In the chart, NI is number of interfaces, CL is chain length, NI : CL is the ratio of number of interfaces to chain length, and PA is percent active residues.

Table S3 A confusion matrix of correlations between all features.

Table S4 Result of inclusion of complexes created from BIOMT field of pdb file.

5 Material Available on Request

Trained models for new predictions, scores of individual proteins for every test conducted, etc. are available upon request.

Author details
[1] School of Computer Science, McGill, Montreal, CA. [2] Department of Microbiology and Immunology, McGill, Montreal, CA. [3] Department of Anatomy and Cell Biology, McGill, Montreal, CA. [4] Department of Biology, McGill, Montreal, CA. [5] Department of Mathematics and Statistics, McGill, Montreal, CA. [6] Department of Biology and Computer Science, McGill, Montreal, CA. [7] Department of Physiology, McGill Montreal, CA.

References

1. Sanner, M.F., Olson, A.J., Spehner, J.-C.: Reduced surface: an efficient way to compute molecular surfaces. Biopolymers **38**(3), 305–320 (1996)
2. Pettersen, E.F., Goddard, T.D., Huang, C.C., Couch, G.S., Greenblatt, D.M., Meng, E.C., Ferrin, T.E.: Ucsf chimera: a visualization system for exploratory research and analysis. Journal of computational chemistry **25**(13), 1605–1612 (2004)
3. Pintar, A., Carugo, O., Pongor, S.: Cx, an algorithm that identifies protruding atoms in proteins. Bioinformatics **18**(7), 980–984 (2002)
4. Pettit, F.K., Bowie, J.U.: Protein surface roughness and small molecular binding sites. Journal of molecular biology **285**(4), 1377–1382 (1999)
5. Fauchere, J., Pliska, V.: Hydrophobic parameters pi of amino-acid side chains from the partitioning of n-acetyl-amino-acid amides. Eur. J. Med. Chem **18**(3), 369–375 (1983)
6. Sillerud, L.O., Larson, R.S.: Design and structure of peptide and peptidomimetic antagonists of protein-protein interaction. Current Protein and Peptide Science **6**(2), 151–169 (2005)
7. Baker, N.A., Sept, D., Joseph, S., Holst, M.J., McCammon, J.A.: Electrostatics of nanosystems: application to microtubules and the ribosome. Proceedings of the National Academy of Sciences **98**(18), 10037–10041 (2001)
8. Fernandez-Recio, J., Totrov, M., Skorodumov, C., Abagyan, R.: Optimal docking area: a new method for predicting protein–protein interaction sites. PROTEINS: Structure, Function, and bioinformatics **58**(1), 134–143 (2005)
9. Coleman, R.G., Burr, M.A., Souvaine, D.L., Cheng, A.C.: An intuitive approach to measuring protein surface curvature. Proteins: Structure, Function, and Bioinformatics **61**(4), 1068–1074 (2005)
10. Pupko, T., Bell, R.E., Mayrose, I., Glaser, F., Ben-Tal, N.: Rate4site: an algorithmic tool for the identification of functional regions in proteins by surface mapping of evolutionary determinants within their homologues. Bioinformatics **18**(suppl 1), 71–77 (2002)
11. Valdar, W.S.: Scoring residue conservation. Proteins: Structure, Function, and Bioinformatics **48**(2), 227–241 (2002)
12. Yang, Z.R., Thomson, R., McNeil, P., Esnouf, R.M.: Ronn: the bio-basis function neural network technique applied to the detection of natively disordered regions in proteins. Bioinformatics **21**(16), 3369–3376 (2005)
13. Ashburner, M., Ball, C.A., Blake, J.A., Botstein, D., Butler, H., Cherry, J.M., Davis, A.P., Dolinski, K., Dwight, S.S., Eppig, J.T., Harris, M.A., Hill, D.P., Issel-Tarver, L., Kasarskis, A., Lewis, S., Matese, J.C., Richardson, J.E., Ringwald, M., Rubin, G.M., Sherlock, G.: Gene ontology: tool for the unification of biology. Nature Genetics **25**, 25–29 (2000)
